# Association between haemodynamics during cardiopulmonary resuscitation and cerebral arterial enhancement

**DOI:** 10.1016/j.resplu.2026.101338

**Published:** 2026-04-21

**Authors:** Yasuaki Koyama, Tasuku Matsuyama, Koki Nakada, Saki Morikawa, Kaoru Fujisawa, Maiko Motoki, Yuji Takahashi, Hideki Hashimoto

**Affiliations:** a Department of Emergency and Critical Care Medicine, Hitachi General Hospital, Hitachi City, Ibaraki, Japan; b Department of Emergency Medicine, Kyoto Prefectural University of Medicine, Kyoto City, Kyoto, Japan

**Keywords:** Cardiopulmonary resuscitation, Mean arterial–venous pressure gradient, Cerebral perfusion, Contrast-enhanced computed tomography, Haemodynamics

## Abstract

**Aim:**

The extent to which antegrade or retrograde blood flow occurs during cardiopulmonary resuscitation (CPR) remains unclear. We investigated the relationship between arterial–venous pressure conditions during CPR and cerebral arterial enhancement on contrast-enhanced computed tomography (CT) to identify haemodynamic parameters associated with antegrade cerebral blood flow.

**Methods:**

This prospective observational single-centre study included adult patients with non-traumatic out-of-hospital cardiac arrest who did not achieve return of spontaneous circulation and underwent contrast-enhanced CT after CPR termination. Femoral arterial and venous pressures were continuously recorded during LUCAS 3-assisted chest compressions. Analysed parameters included arterial systolic (A sys), mean (A mean), diastolic (A dias) pressures, and arterial–venous pressure gradients (ΔSys A–V and ΔMean A–V). Contrast enhancement of the circle of Willis was evaluated.

**Results:**

Among 38 patients, 11 (29%) demonstrated contrast enhancement of the circle of Willis. Higher A sys, A mean, ΔSys A–V, and ΔMean A–V were significantly associated with cerebral arterial enhancement (all *p* < 0.001). The proportion of patients with arterial enhancement increased with higher ΔMean A–V, and all patients with ΔMean A–V ≥ 20 mmHg showed complete arterial enhancement. Venous enhancement from the superior sagittal sinus to the inferior vena cava was observed in nearly all patients.

**Conclusions:**

Higher arterial pressures and greater arterial–venous pressure gradients were associated with cerebral arterial enhancement during CPR, suggesting that a mean arterial–venous pressure gradient may be associated with antegrade cerebral perfusion and represent a physiological target for optimising resuscitation.

## Highlights

• Arterial systolic and mean pressure during CPR correlated with cerebral enhancement.
• Arterial–venous pressure gradients during CPR correlated with cerebral enhancement.
• Larger ΔMean A-V values indicated more antegrade blood flow.
• ΔMean A-V may be important for antegrade perfusion during CPR.
• ΔMean A-V values may represent a hypothesis-generating haemodynamic marker.

## Introduction

The goal of cardiopulmonary resuscitation (CPR) is to ensure the circulation of oxygenated blood throughout the body, particularly to maintain adequate cerebral perfusion and improve neurological outcomes. High-quality chest compressions (CCs) are fundamental to achieve this goal. However, current guidelines uniformly define compression depth, rate, and hand position without accounting for anatomical differences, such as chest wall thickness or cardiac position,^1^, ^2^ and may therefore fail to ensure optimal circulation in every patient. Although end-tidal carbon dioxide (EtCO_2_) and diastolic arterial pressure are objective indicators of CC quality, they have inherent limitations. EtCO_2_ is strongly influenced by the aetiology of cardiac arrest (CA) and medication effects.^3^ Although diastolic arterial pressure correlates with the likelihood of return of spontaneous circulation (ROSC) as a surrogate for coronary perfusion pressure,^4^, ^5^ whether it directly reflects systemic or cerebral perfusion remains unclear.

Furthermore, the haemodynamic mechanism underlying CCs is not fully understood. Traditionally, CCs are thought to generate predominantly antegrade blood flow,^6^ however, retrograde flow into the jugular veins and inferior vena cava has also been reported.^7^ Blood flow is driven by the gradient between arterial and venous pressures,^8^ and various arterial–venous pressure patterns have been observed during CCs.^9^ Notably, the difference between arterial and venous mean pressures was strongly associated with the achievement of ROSC.^10^ However, the extent to which antegrade or retrograde blood flow occurs during CCs remains unclear.

Therefore, we aimed to clarify the relationship between antegrade blood flow and the arterial–venous pressure conditions during CCs and propose a novel indicator for evaluating cerebral and systemic perfusion.

## Methods

### Study design

This prospective observational study was conducted at a single tertiary emergency department (ED) in Japan between December 2023 and April 2025. The study was approved by the Institutional Review Board (IRB) of Hitachi General Hospital (IRB number: 2022-36; Date: August 29, 2022). All research procedures complied with the ethical standards of the responsible human research committee and principles of the 1975 Declaration of Helsinki. To facilitate advances in resuscitation science involving unconscious patients, the US Food and Drug Administration provides specific provisions allowing a waiver of informed consent in emergency medical research. Accordingly, the IRB granted a waiver of informed consent that applied irrespective of patient survival or neurological recovery.

All data present in this article were anonymised; therefore, publication consent was not required.

### Study participants

We enrolled patients >15 years who experienced non-traumatic out-of-hospital cardiac arrest (OHCA), had arterial and venous pressures recorded during CPR, and underwent contrast-enhanced computed tomography (CT) after CPR termination. At our institution, when the cause of cardiac arrest remained unclear based on the history of present illness, contrast-enhanced CT was performed to investigate the underlying cause as soon as the CT suite became available (eFig. 1). Patients who had a do-not-attempt-resuscitation order, achieved ROSC, received extracorporeal CPR, did not undergo pressure measurement, or whose cause of CA had already been identified were excluded.

### CPR protocol

CPR was performed after ED arrival in accordance with the 2020 resuscitation guidelines.^1^ After intubation, patients were connected to a ventilator set to a tidal volume of 400–600 mL, positive end-expiratory pressure of 4–6 cm H_2_O, and respiratory rate of 20 breaths/min. Manual or mechanical CCs were initially performed asynchronously,^1^ and were transitioned to the LUCAS 3 system (LUCAS™ 3 Chest Compression System, JOLIFE AB Inc., Lund, Sweden), which was used for at least 2 min before CPR termination. As soon as possible after ED arrival, a 4-Fr introducer sheath (Radifocus Introducer Ⅱ H, Terumo Corp., Tokyo, Japan) was placed immediately in the femoral artery and vein using the Seldinger technique under ultrasound guidance. Arterial and venous pressures were recorded continuously and simultaneously, and catheter placement was confirmed by contrast-enhanced CT after CPR termination (eFig. 1).

### Contrast-enhanced CT

**Fig. 1:**
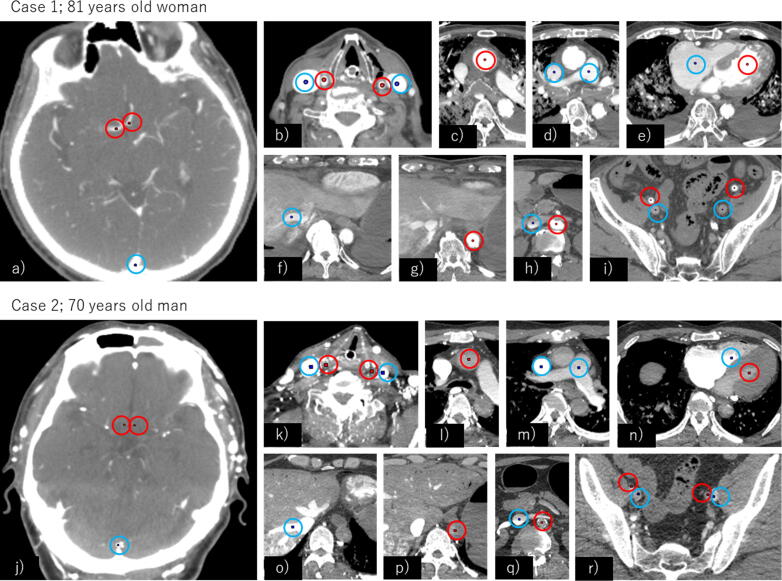
**Contrast-enhanced CT of 20 target blood vessels in two representative cases**. Red circles indicate arteries, and blue circles indicate veins. Red and blue points denote regions of interest in which CT attenuation value (HU) was measured (a/i/j/r; φ1 mm, b–h/k–q; φ3 mm). a/j. Bilateral anterior cerebral arteries in the circle of Willis and the superior sagittal sinus b/k. Bilateral common carotid arteries and internal jugular veins at the level of thyroid cartilage c/l. Ascending aorta at the level of tracheal bifurcation d/m. Pulmonary artery trunk and superior vena cava at the level of pulmonary artery bifurcation e/n. Left and right ventricles f/o. Hepatic vein at the junction with the inferior vena cava g/p. Descending aorta at the level of the diaphragm h/q. Descending aorta and inferior vena cava at the level of the left renal vein i/r. Bilateral external iliac arteries and veins immediately proximal to the sheath tips.

In accordance with previous reports,^11^ contrast-enhanced CT was performed during CCs with concurrent contrast agent infusion. An automatic injector (Dual Shot GX, Nemoto Kyorindo Inc., Japan) was connected to a peripheral venous catheter placed in either upper limb during CPR, and 100 mL of contrast medium (iopamidol [Oypalomin 300 Injection Syringe, Konica Minolta Holdings Inc., Japan], a non-ionic agent commonly used in clinical practice) was administered at a rate of 1 mL/s.^11^ CCs, performed using the LUCAS 3 system, were initiated simultaneously with contrast administration and continued for 2 min. Contrast-enhanced CT imaging was performed using a 64-channel multidetector-row CT scanner (SCENARIA View; FUJIFILM Healthcare Corp., Tokyo, Japan). Imaging from the head to the pelvis was acquired using a helical protocol with the following parameters: automatic mA (300–600; standard deviation, 6.0), 120 kV, rotation time of 0.5 s, collimation of 0.625 mm, a pitch of 0.8, a table speed of 13.75 mm per rotation, and a reconstructed slice thickness of 2.5 mm. CT attenuation values were obtained from the lumens of 20 vascular structures: the bilateral anterior cerebral arteries in the circle of Willis and the superior sagittal sinus (Fig. 1a/j); bilateral common carotid arteries and internal jugular veins at the level of the thyroid cartilage (Fig. 1b/k); ascending aorta at the level of tracheal bifurcation (Fig. 1c/l); pulmonary artery trunk and superior vena cava at the level of pulmonary artery bifurcation (Fig. 1d/m); both ventricles (Fig. 1e/n); hepatic vein at the junction with the inferior vena cava (Fig. 1f/o); descending aorta at the level of the diaphragm (Fig. 1g/p); descending aorta and inferior vena cava at the level of the left renal vein (Fig. 1h/q); and bilateral external iliac arteries and veins immediately proximal to the sheath tips (Fig. 1i/r).

A circular region of interest (ROI) with a diameter of 3 mm was placed at the centre of each target vessel. For the bilateral anterior cerebral arteries, superior sagittal sinus, and bilateral external iliac arteries and veins, the ROI diameter was reduced to 1 mm, consistent with prior studies.^11^ A threshold of 140 Hounsfield units (HU) was used as the minimum level of intravascular enhancement required for analysis.^11^, ^12^, ^13^

### Data collection

We collected data on patient demographics, CA location, witness status, bystander CPR, initial cardiac rhythms recorded by emergency medical technicians (EMTs) and on ED arrival, the interval from last confirmed survival to CPR initiation, dispatch-to-EMT and dispatch-to-ED arrival times, CPR duration, and the time from CPR termination to contrast-enhanced CT. During CPR, femoral arterial and venous pressure waveforms were continuously monitored. For each CC, systolic and diastolic pressures from both vessels were recorded for analysis.

### Outcome measures

The primary outcome was the association between arterial and venous pressure indices during the final 30 s of CCs and the presence or absence of contrast enhancement in major arteries and veins, including the circle of Willis.

### Data analysis

**Fig. 2:**
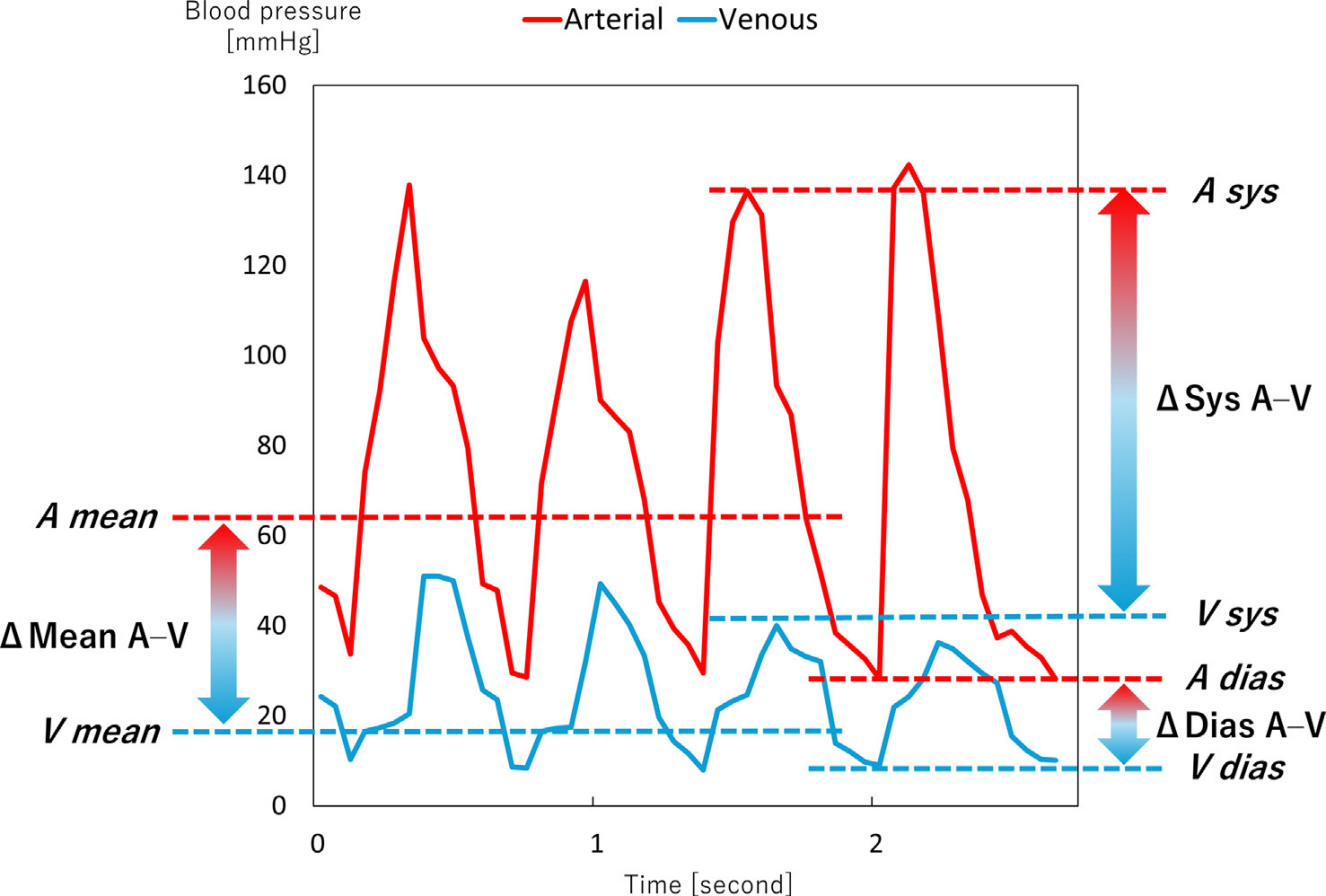
**Hemodynamic variables from pressure waveforms of the femoral artery and vein during CPR**. A sys, arterial systolic pressure (corresponding to the compression phase of chest compressions); A dias, arterial diastolic pressure (corresponding to the decompression phase); A mean, arterial mean pressure (=A dias + 1/3 [A sys – A dias]). V sys, venous systolic pressure (compression phase); V dias, venous diastolic pressure (decompression phase); V mean, venous mean pressure (=V dias + 1/3 [V sys – V dias]); ΔSys A–V, systolic arterial–venous pressure gradient; ΔDias A–V; diastolic arterial–venous pressure gradient; ΔMean A–V, mean arterial–venous pressure gradient.

Data were summarised using medians or proportions, depending on variable type. The arterial parameters analysed included arterial systolic pressure (A sys, corresponding to the compression phase of CCs), arterial diastolic pressure (A dias, corresponding to the decompression phase), and arterial mean pressure (A mean = A dias + 1/3 [A sys − A dias]). Venous parameters included venous systolic pressure (V sys, compression phase), venous diastolic pressure (V dias, decompression phase), and venous mean pressure (V mean = V dias + 1/3 [V sys − V dias]). In addition, systolic (ΔSys A–V), diastolic (ΔDias A–V), and mean (ΔMean A–V) arterial–venous pressure gradients were analysed (Fig. 2).

For each patient, systolic and diastolic pressures were identified for every CC, and mean values were calculated by averaging all measurements obtained during the final 30-s CC period. These time-averaged values were then compared between patients with and without contrast enhancement of the circle of Willis. Results are presented as medians. Given the exploratory evaluation of multiple haemodynamic parameters, the nine variables presented in Table 2 were considered a single family of tests, and Bonferroni correction was applied. A *p*-value < 0.0056 (0.05/9) was considered statistically significant. The area under the curve (AUC) was also calculated for each parameter. Parameters showing significant differences were further stratified in 10-mmHg increments, and a heatmap was generated based on the proportion of patients demonstrating contrast enhancement in each vessel. For each vessel, changes in the proportion of patients with contrast enhancement across increasing parameter values were evaluated using the Cochran–Armitage test. Statistical significance was defined as *p* < 0.05, two sided. For the vessel-level trend analyses of contrast enhancement based on haemodynamic parameters, Bonferroni correction was applied to account for multiple comparisons. Statistical significance was defined as *p* < 0.0025 (0.05/20). Sensitivity analyses were also performed over the 2-min CC period. Analyses were performed using EZR software (Saitama Medical Centre, Jichi Medical University, Saitama, Japan),^14^ and heatmap visualisations were generated using R version 4.5.1 (R Foundation for Statistical Computing, Vienna, Austria).^15^

## Results

**Fig. 3:**
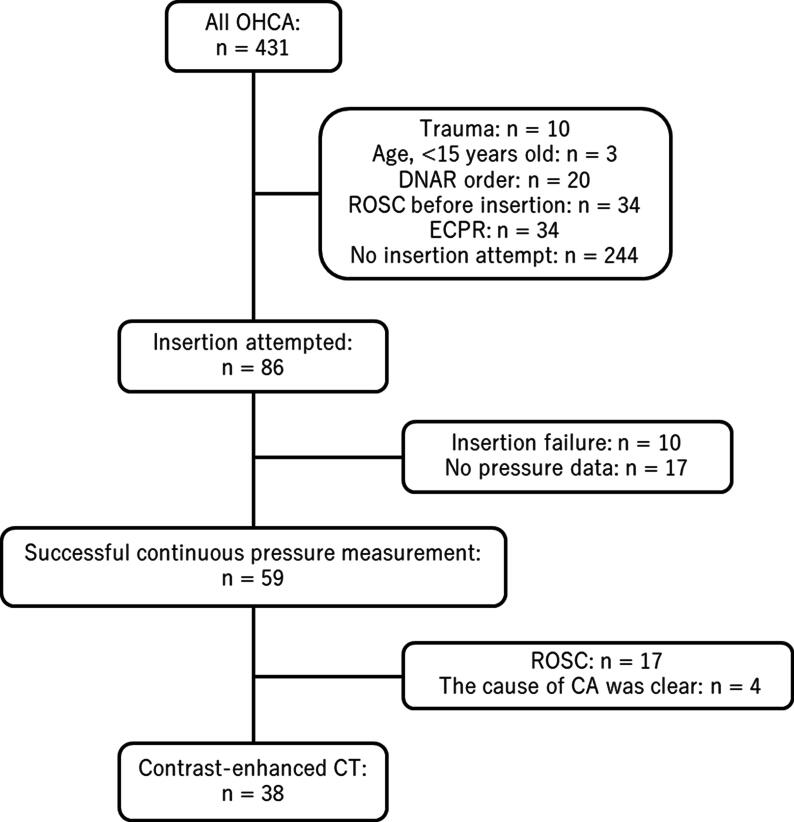
**Flow chart of patient selection in this study**. CA, cardiac arrest; CT, computed tomography; DNAR, do not attempt resuscitation; ECPR, extracorporeal cardiopulmonary resuscitation; OHCA, out-of-hospital cardiac arrest; ROSC, return of spontaneous circulation.

Of 431 patients with OHCA were transported to the hospital by ambulance, 86 were enrolled, and continuous pressure measurements were successfully recorded in 59 patients. Among these, 42 did not achieve ROSC. The cause of CA was clearly identified in 4 patients before CT; therefore, 38 patients (males, *n* = 23; median age, 79 years; Table 1) who underwent contrast-enhanced CT were included in the final analysis (Fig. 3). No significant differences were observed in baseline characteristics between patients with and without contrast enhancement of the circle of Willis (Table 1).

**Table 1:** Patient characteristics.

	**Total**	**Contrast-enhanced**	**No contrast-enhanced**
Number of individuals	38	11	27
Age (years)	79.0 [71.5–83.8]	81.0 [77.0–83.5]	78.0 [70.5–83.5]
Male sex	23 (60.5)	4 (36.4)	19 (70.4)
Cardiac arrest at home/residency area	34 (89.5)	10 (90.9)	24 (88.9)
Witnessed arrest	9 (23.7)	2 (18.2)	7 (25.9)
Bystander CPR	14 (36.8)	3 (27.3)	11 (40.7)
**Initial cardiac rhythm on EMT arrival**			
Vf/pulseless VT	1 (2.6)	0 (0.0)	1 (3.7)
PEA	7 (18.4)	2 (18.2)	5 (18.5)
Asystole	30 (78.9)	9 (81.8)	21 (77.8)
**Initial cardiac rhythm at the ED**			
Vf/pulseless VT	0 (0.0)	0 (0.0)	0 (0.0)
PEA	12 (31.6)	3 (27.3)	9 (33.3)
Asystole	26 (68.4)	8 (72.7)	18 (66.7)
Time from last confirmed survival to CPR initiation (min)	62.5 [26.3–171.3]	71.0 [32.5–103.5]	44.0 [20.0–225.0]
Time from dispatch to first arrival of EMT (min)	8.0 [6.0–9.8]	9.0 [6.0–14.0]	8.0 [6.5–9.0]
Time from dispatch to arrival at the ED (min)	37.0 [30.0–48.3]	40.0 [27.5–46.0]	37.0 [30.5–47.5]
CPR duration (min)	64.0 [58.0–78.5]	60.0 [55.5–72.0]	64.0 [59.0–79.5]
Prehospital CPR duration by EMT (min)	28.0 [23.0–39.8]	28.0 [18.5–37.0]	28.0 [23.5–40.0]
Total time for pressure measurement	16.5 [15.6–18.6]	16.1 [15.9–17.9]	17.0 [15.4–18.6]
Time during LUCAS 3-assisted chest compressions	3.7 [2.2–5.5]	3.2 [2.6–6.3]	3.7 [2.1–5.0]
Time from CPR termination to contrast-enhanced CT (min)	27.0 [25.0–36.5]	30.0 [26.5–36.0]	27.0 [23.5–34.5]
**Cause of cardiac arrest**			
Cardiogenic	7 (18.4)	4 (36.4)	3 (11.1)
Ascending aortic dissection	7 (18.4)	3 (27.3)	4 (14.8)
Respiratory	5 (13.2)	2 (18.2)	3 (11.1)
Aortic rupture	2 (5.3)	1 (9.1)	1 (3.7)
Subarachnoid haemorrhage	1 (2.6)	0 (0.0)	1 (3.7)
Pulmonary embolism	1 (2.6)	0 (0.0)	1 (3.7)
Others	5 (13.2)	0 (0.0)	5 (18.5)
Unknown	10 (26.3)	1 (9.1)	9 (33.3)

Data are presented as median [interquartile range], or *n* (%).

CPR, cardiopulmonary resuscitation; CT, computed tomography; ED, emergency department; EMT, emergency medical technician; PEA, pulseless electrical activity; Vf, ventricular fibrillation; VT, ventricular tachycardia.

The median A sys, A mean, and A dias were 51.1, 22.3, and 6.7 mmHg, respectively. Significant differences were observed between patients with and without contrast enhancement of the circle of Willis in A sys (median, 101.8 mmHg vs. 47.3 mmHg; *p* < 0.001), A mean (36.4 mmHg vs. 17.6 mmHg; *p* < 0.001), ΔSys A–V (36.0 mmHg vs. −2.2 mmHg; *p* < 0.001), and ΔMean A–V (17.8 mmHg vs. −1.4 mmHg; *p* < 0.001). However, the AUCs for A sys, A mean, ΔSys A–V, and ΔMean A–V were all greater than 0.8 (Table 2).

**Table 2:** Comparison of the values during the final 30 s of chest compressions with the LUCAS 3 device between patients with and without enhancement of the circle of Willis.

	**Last 30 s**	**Contrast-enhanced**	**No Contrast-enhanced**	***P*-value**	**AUC (95%CI)**
A sys	51.1 [33.5–76.3]	101.8 [70.4–110.8]	47.3 [26.5–54.2]	<0.001	0.88 (0.76–1.00)
A mean	22.3 [15.2–32.4]	36.4 [30.2–54.5]	17.6 [11.0–23.7]	<0.001	0.88 (0.77–1.00)
A dias	6.7 [2.8–11.4]	10.3 [6.1–18.7]	6.0 [1.1–9.4]	0.045	0.71 (0.51–0.91)
V sys	41.6 [30.5–61.6]	46.0 [39.2–67.2]	36.7 [27.9–58.0]	0.159	0.65 (0.47–0.83)
V mean	17.5 [13.5–25.6]	23.1 [16.2–29.0]	16.2 [12.7–25.6]	0.251	0.62 (0.44–0.80)
V dias	6.8 [3.4–11.2]	9.3 [2.0–10.8]	6.7 [4.0–10.9]	0.727	0.54 (0.31–0.77)
ΔSys A–V	6.1 [−4.7–30.2]	36.0 [15.0–75.4]	−2.2 [−16.5–9.7]	<0.001	0.87 (0.76–0.99)
ΔMean A–V	0.5 [−4.4–10.0]	17.8 [2.9–30.6]	−1.4 [−6.7–4.2]	<0.001	0.82 (0.66–0.97)
ΔDias A–V	−1.3 [−5.6–2.7]	5.4 [−6.5–9.5]	−1.9 [−5.2–0.8]	0.213	0.63 (0.38–0.88)

Data are presented as median [interquartile range] (mmHg).

AUC, area under the curve; A dias, arterial diastolic pressure; A mean, arterial mean pressure; A sys, arterial systolic pressure; CI, confidence interval; V dias, venous diastolic pressure; V mean, venous mean pressure; V sys, venous systolic pressure; ΔDias A–V, the difference between arterial and venous diastolic pressure; ΔMean A–V, the difference between arterial and venous mean pressures; Δsys A–V, the difference between arterial and venous systolic pressure.

**Fig. 4:**
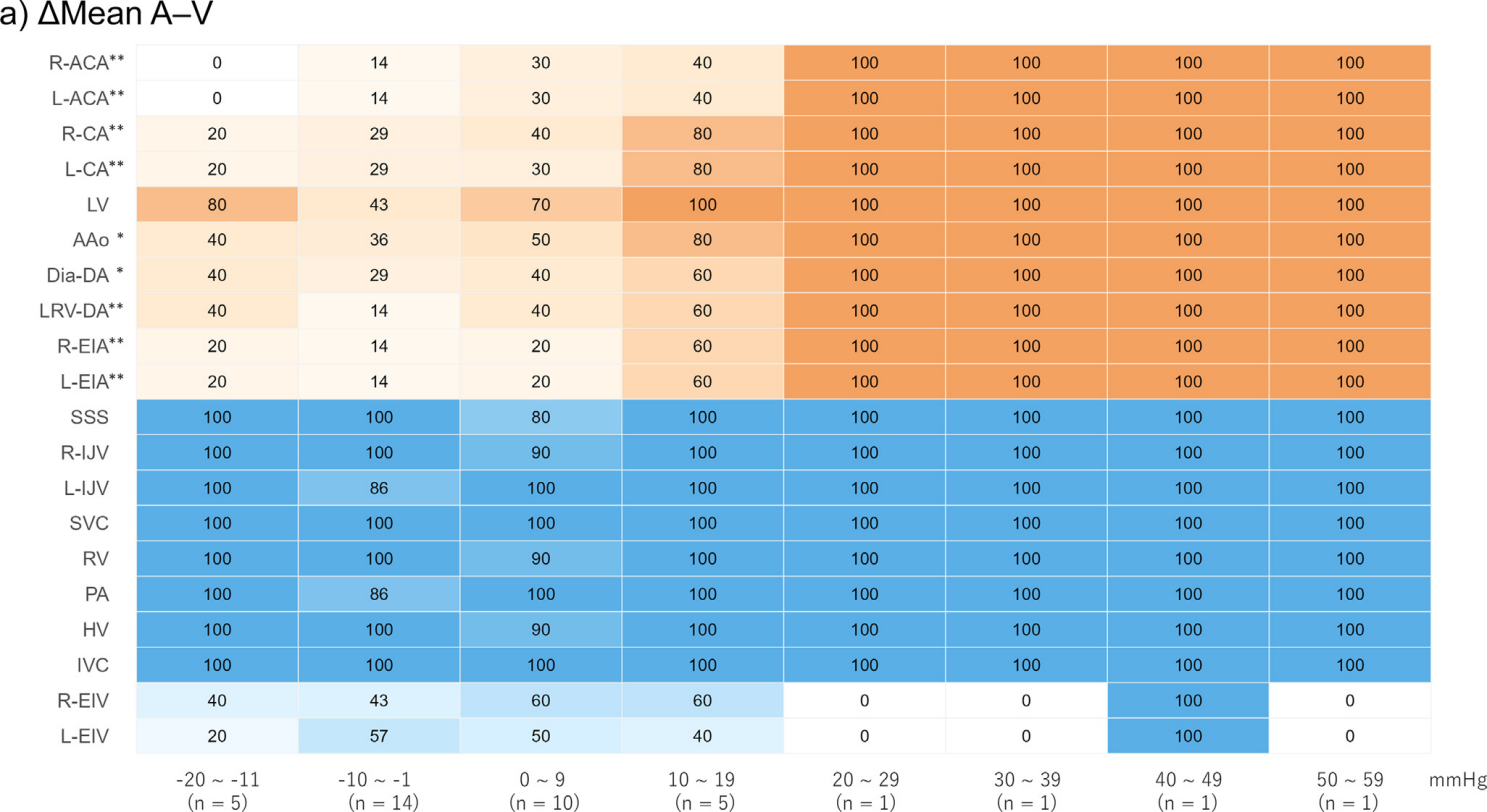

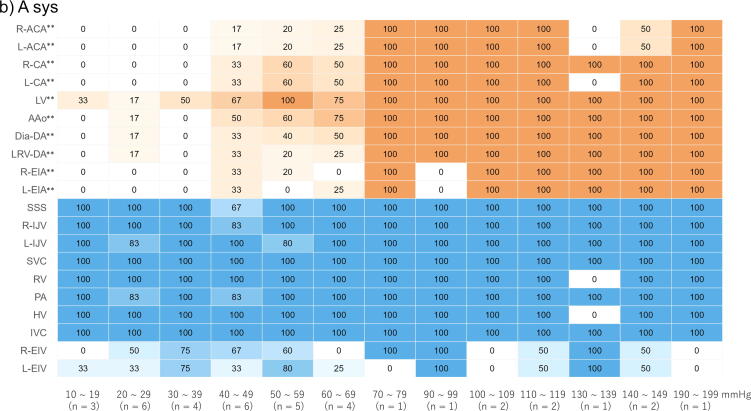
**Heatmap analysis of the final 30 s of each parameter obtained during chest compression performed with the LUCAS 3 device**. (a) ΔMean A–V; (b) A sys. The remaining figures (eFig. 1a–c) are provided in the Supplementary File. Data are presented as the proportion (%) of patients with contrast enhancement in each vessel. *P* values were calculated using the Cochran–Armitage test for trend (*p for trend <0.05; **p for trend <0.01). A sys, arterial systolic pressure; ΔMean A–V, difference between arterial and venous mean pressures. R-ACA, right-anterior cerebral artery; L-ACA, left-anterior cerebral artery; R-CA, right-common carotid artery; L-CA, left-common carotid artery; LV, left ventricle; AAo, ascending aorta; Dia-DA, descending aorta at the level of the diaphragm; LRV-DA, descending aorta at the level of the left renal vein; R-EIA, right-external iliac artery; L-EIA, left-external iliac artery; SSS, superior sagittal sinus; R-IJV, right-internal jugular vein; L-IJV, left-internal jugular veins; SVC, superior vena cava; RV, right ventricle; PA, pulmonary artery; HV, hepatic vein; IVC, inferior vena cava at the level of the left renal vein; R-EIV, right-external iliac vein; L-EIV, left-external iliac vein.

Heatmap analysis revealed that higher ΔMean A–V values were significantly associated with an increased proportion of patients showing enhancement in the arterial system, including the bilateral anterior cerebral arteries in the circle of Willis, bilateral common carotid arteries, ascending aorta, descending aorta, and bilateral external iliac arteries (*p* < 0.05). Enhancement pattern of the circle of Willis exhibited a clear upward trend with increasing ΔMean A–V, and all patients with a ΔMean A–V ≥ 20 mmHg demonstrated arterial system enhancement (Fig. 4). Higher values of A sys, A mean, ΔSys A–V, and ΔMean A–V were also associated with an increasing proportion of patients exhibiting enhancement in the circle of Willis (*p* for trend < 0.0025); however, even at higher values of these parameters, some patients showed no enhancement in the circle of Willis (Fig. 4 and eFig. 2 in the Supplementary File). In contrast, the venous system, from the superior sagittal sinus to the inferior vena cava, was enhanced in almost all patients, even in the absence of arterial system enhancement.

Sensitivity analyses performed during the 2-min period of CCs yielded results consistent with those of the primary analysis (eTable and eFig. 3 in the Supplementary File).

## Discussion

Our findings revealed that A sys, A mean, ΔSys A–V, and ΔMean A–V during CPR were significantly associated with contrast enhancement of the circle of Willis, which represents the major cerebral arteries. Higher values of these parameters were associated with an increased proportion of patients with arterial system enhancement, and the enhancement pattern of the circle of Willis increased progressively with rising ΔMean A–V.

Cerebral perfusion pressure (CPP) is calculated as mean arterial pressure (MAP) minus either intracranial pressure (ICP) or central venous pressure (CVP), whichever is higher.^16^ Because MAP is used in this calculation, although diastolic pressure contributes, an increase in systolic arterial pressure elevates MAP and, consequently, CPP. Animal studies have revealed that complete chest recoil (100%), compared with partial recoil (75%), reduces right atrial and venous pressures, increases systolic, mean, and diastolic arterial pressures, and enhances CPP.^17^ In a porcine model, MAP reduction during CCs resulted in decreased aortic and carotid blood flow.^18^ Other experimental data showed that compressing the left ventricle rather than the standard sternal position increased arterial pressure and cerebral blood flow, with systolic pressure and MAP exhibiting greater increases than diastolic pressure.^19^ Collectively, these findings indicate that systolic arterial pressure and MAP play a crucial role in maintaining antegrade cerebral perfusion during CPR.

Our analysis revealed that higher ΔMean A–V values were associated with an increased proportion of patients with arterial system enhancement, and when ΔMean A–V exceeded 20 mmHg, all arteries were enhanced in all patients. Similarly, higher A sys, A mean, and ΔSys A–V values were associated with arterial enhancement; however, some patients showed no enhancement of the circle of Willis even at high values. Since antegrade blood flow is driven by the pressure gradient between the arterial and venous systems,^8^ a greater pressure difference is thought to promote antegrade blood flow throughout the body, including the brain. Conversely, even when systolic arterial pressure is high, elevated venous pressure can reduce the pressure difference, resulting in a negative pressure gradient.^8^, ^9^ Recently, reduced arterial pressure and elevated venous pressure have been recognised as contributing factors to organ dysfunction in cardiorenal syndrome.^20^ Similarly, mean perfusion pressure (MPP = MAP − CVP) has been associated with acute kidney injury,^21^ and the arterial–venous pressure gradient has increasingly been recognised as an important determinant of organ perfusion. In the previous study, a larger ΔMean A–V was also associated with a higher probability of achieving ROSC.^10^ In the present study, the absence of cerebral enhancement in some cases despite high A sys values suggests that the mean arterial–venous pressure gradient may be more related to cerebral perfusion than systolic arterial pressure alone.

In this study, the venous system was contrast-enhanced from the superior sagittal sinus to the inferior vena cava in almost all patients. Retrograde venous flow during CPR observed on postmortem CT has been reported previously.^7^, ^22^ Contrast enhancement from the superior sagittal sinus to the inferior vena cava occurred regardless of arterial pressure, suggesting the presence of retrograde venous flow induced by CCs. Additionally, in cases with a large ΔMean A–V, both the bilateral external iliac arteries and their corresponding veins were enhanced, implying anterograde passage of contrast from arteries to veins. Despite the potential influence of the automatic injector, retrograde blood flow occurred in almost all patients even when CCs were performed using the LUCAS device, which provides consistent-quality compressions.

Recently, diastolic arterial pressure has been emphasised in current guidelines,^21^ whereas systolic arterial pressure and MAP during resuscitation have also gained increasing attention.^23^, ^24^, ^25^ Our findings suggest that effective resuscitation circulation should be evaluated by considering both arterial and venous pressures rather than arterial pressure alone. The pressure gradients were associated with arterial contrast enhancement during CPR and may represent a hypothesis-generating haemodynamic marker warranting validation in prospective studies. Therefore, applying customised or individualised CPR rather than a uniform standard CPR is important. Real-time assessment of cerebral blood flow using non-invasive monitoring modalities, such as near-infrared spectroscopy, would be ideal, as direct measurement of arterial and venous pressures is highly invasive.^26^, ^27^, ^28^ CPR achieving a higher systemic perfusion pressure appeared to improve cerebral circulation and brain recovery. These findings require further investigation and may contribute to the development of future resuscitation strategies.

This study has several limitations. First, as it included only patients who did not achieve ROSC, cerebral blood flow during CCs in patients who attained ROSC or favourable neurological outcomes remains unknown. Larger studies that include patients with good neurological recovery are required to elucidate these differences. Second, approximately 30 min elapsed from CPR termination to contrast-enhanced CT (Table 1); thus, the arterial and venous pressures analysed in this study were not those measured during LUCAS 3-assisted CCs at the time of CT acquisition. Because adrenaline was administered during resuscitation, arterial pressures during CPR may have been relatively higher than during CT scan. Although the compression position and technique were unchanged, progressive circulatory deterioration over the 30-min interval may have resulted in lower arterial pressures at the time of CT. Although enhancement observed later cannot directly confirm cerebral perfusion during CPR, preserved vascular reactivity^29^, ^30^ and the absence of early thrombotic occlusion^31^, ^32^ suggest that the enhancement patterns observed on CT may be partially related to the preceding haemodynamic conditions at CPR termination. Nevertheless, because visualisation of the circle of Willis on contrast-enhanced CT required a ΔMean A–V ≥ 20 mmHg approximately 30 min earlier, this pressure gradient value is likely related to cerebral perfusion during resuscitation. Third, arterial and venous pressures were obtained from the femoral vessels, which may not accurately represent the pressure gradients between the cerebral arteries, cerebral veins, and ICP. An animal study has shown that mean intracranial arterial pressure is approximately 20 mmHg lower than mean descending aortic pressure under normal conditions and about 10 mmHg lower during CPR, and that higher mean intracranial arterial pressure is associated with greater cerebral blood flow.^33^ Given the observational design and the absence of outcome correlation, these findings should be interpreted as hypothesis-generating and require confirmation in larger prospective studies. Fourth, the underlying causes of CA detected on contrast-enhanced CT (such as pulmonary embolism, aortic dissection, or large-vessel stroke) may have affected regional enhancement and acted as confounders. These conditions may have impaired forward flow, and their effects cannot be completely excluded. Finally, the small sample size and potential selection bias may have influenced our findings. Only four patients exhibited a ΔMean A–V ≥ 20 mmHg; therefore, larger studies are needed to confirm the validity of this threshold.

## Conclusions

Higher values of A sys, A mean, ΔSys A–V, and ΔMean A–V during resuscitation were significantly associated with cerebral arterial enhancement. Moreover, a larger ΔMean A–V values were associated with imaging findings consistent with antegrade blood flow, and venous enhancement was observed in almost all patients. Further studies examining haemodynamics during CPR are required to improve neurological outcomes.

## Availability of data and transparency

This study data are retained and archived for a minimum of 3 years after study completion by the IRB of Hitachi General Hospital. All deidentified data generated and analysed during the study are available from the corresponding author upon reasonable request.

## CRediT authorship contribution statement

**Yasuaki Koyama:** Writing – original draft, Validation, Resources, Project administration, Methodology, Investigation, Data curation, Conceptualization. **Tasuku Matsuyama:** Writing – review & editing, Methodology, Formal analysis. **Koki Nakada:** Visualization, Software, Resources, Formal analysis. **Saki Morikawa:** Formal analysis. **Kaoru Fujisawa:** Investigation. **Maiko Motoki:** Investigation, Data curation. **Yuji Takahashi:** Investigation. **Hideki Hashimoto:** Writing – review & editing, Supervision, Project administration, Investigation.

## Funding

This research did not receive any specific grant from funding agencies in the public, commercial, or not-for-profit sectors.

## Declaration of competing interest

The authors declare that they have no known competing financial interests or personal relationships that could have appeared to influence the work reported in this paper.
