# Supplementary material for: Association between haemodynamics during cardiopulmonary resuscitation and cerebral arterial enhancement
Source: Resusc Plus. 2026 Apr 21;29:101338. doi: 10.1016/j.resplu.2026.101338 (PMC13195767; doi:10.1016/j.resplu.2026.101338)
Supplement: Supplementary Data 1 [file mmc1.docx]

**Supplementary File**

**Title:** Association between hemodynamics during cardiopulmonary resuscitation and cerebral arterial enhancement

# eTable – Comparison of the values over 2 min for each parameter obtained with each chest compression using the LUCAS 3 between patients with and without a contrast-enhanced circle of Willis

|  | **2 min** | **Contrast-enhanced** | **No Contrast-enhanced** | **P-value** | **AUC (95%CI)** |
| --- | --- | --- | --- | --- | --- |
| A sys | 51.5 [36.9–75.0] | 91.0 [68.7–117.2] | 48.2 [27.3–53.6] | <0.001 | 0.87 (0.74–0.99) |
| A mean | 22.7 [15.9–30.1] | 37.1 [29.0–48.8] | 18.3 [11.6–24.3] | <0.001 | 0.87 (0.75–0.99) |
| A dias | 7.0 [2.9–11.7] | 9.6 [6.3–19.5] | 5.7 [0.7–9.9] | 0.061 | 0.70 (0.51–0.88) |
| V sys | 42.5 [30.6–62.1] | 49.0 [37.0–67.6] | 40.1 [28.9–58.6] | 0.293 | 0.61 (0.42–0.80) |
| V mean | 18.0 [13.7–26.5] | 23.7 [15.8–28.9] | 17.4 [13.5–26.4] | 0.505 | 0.57 (0.38–0.76) |
| V dias | 7.8 [3.2–11.0] | 10.1 [2.1–10.8] | 7.2 [3.6–11.4] | 0.924 | 0.51 (0.29–0.74) |
| ΔSys A–V | 5.0 [-5.4–28.8] | 34.1 [15.3–74.2] | -1.2 [-14.9–10.8] | <0.001 | 0.87 (0.76–0.98) |
| ΔMean A–V | 0.1 [-3.8–10.2] | 17.3 [1.8–29.1] | -1.0 [-5.7–4.2] | 0.001 | 0.82 (0.66–0.98) |
| ΔDias A–V | -1.3 [-5.0–2.3] | 6.8 [-4.1–10.4] | -1.8 [-4.8–1.3] | 0.124 | 0.66 (0.43–0.90) |
| Data are shown as median [interquartile range] (mmHg).  AUC, area under the curve; A dias, arterial diastolic pressure; A mean, arterial mean pressure; A sys, arterial systolic pressure; CI, confidence interval; V dias, venous diastolic pressure; V mean, venous pressure; V sys, venous systolic pressure; ΔDias A–V, difference between arterial and venous diastolic pressure; ΔMean A–V, difference between arterial and venous mean pressures; Δsys A–V, difference between arterial and venous systolic pressures. | | | | | |

Figures

# eFigure 1. CPR protocol

#
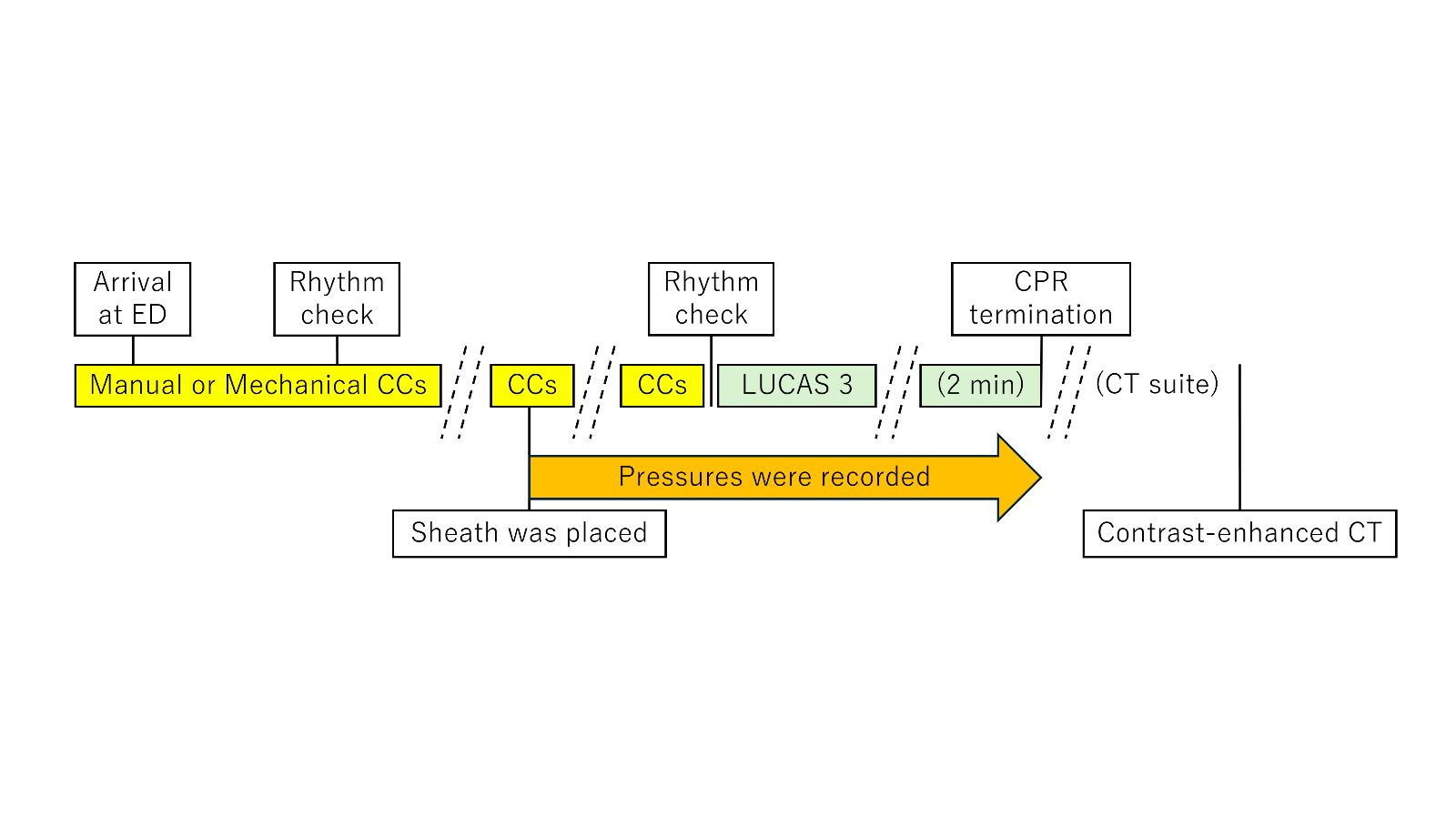


CCs; chest compressions; CPR, cardiopulmonary resuscitation; CT, computed tomography; ED, emergency department.

# eFigure 2. Heatmap analysis of the final 30 seconds of each parameter obtained with each chest compression using the LUCAS 3

1.
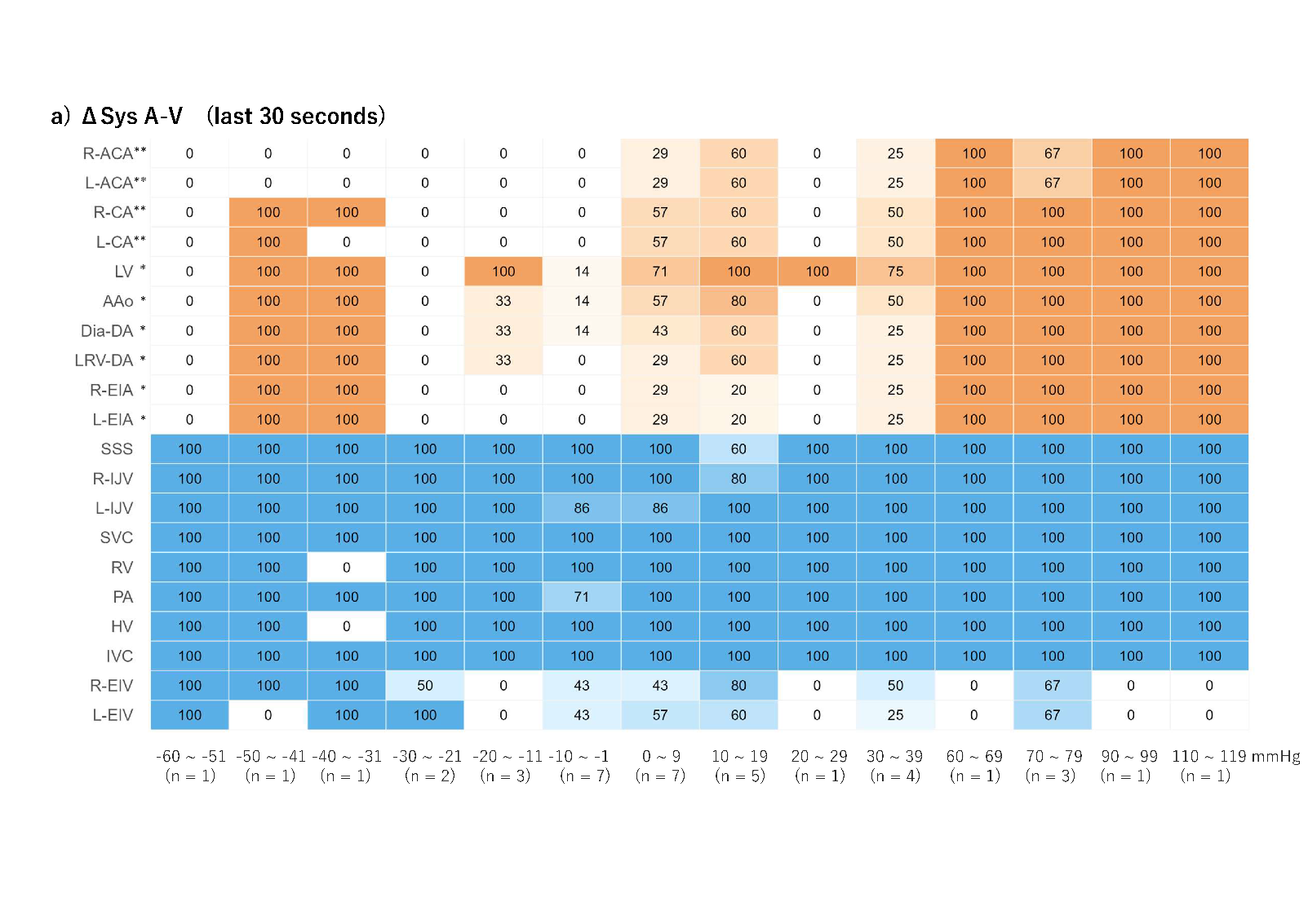
ΔSys A–V

1.
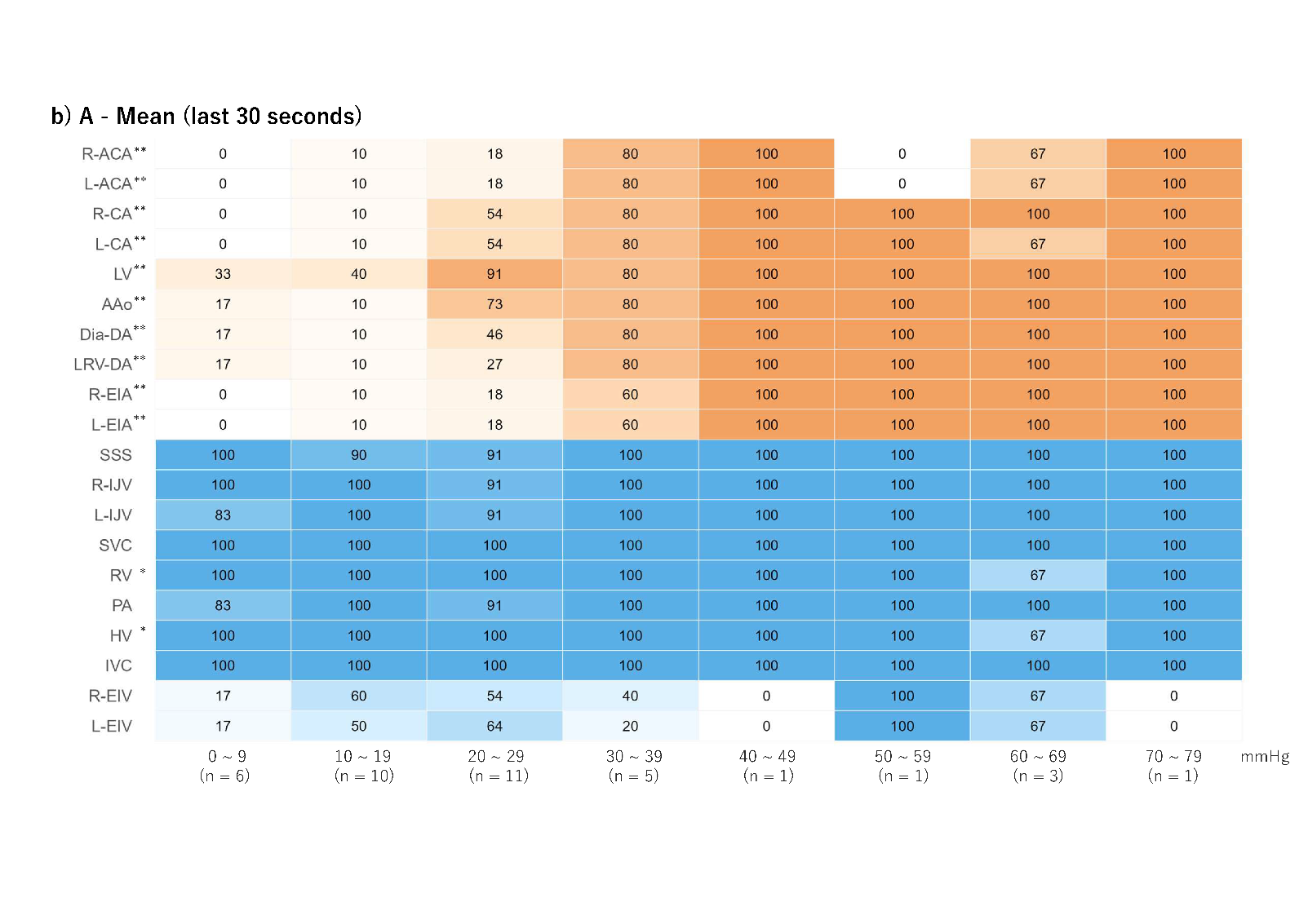
A mean
2.
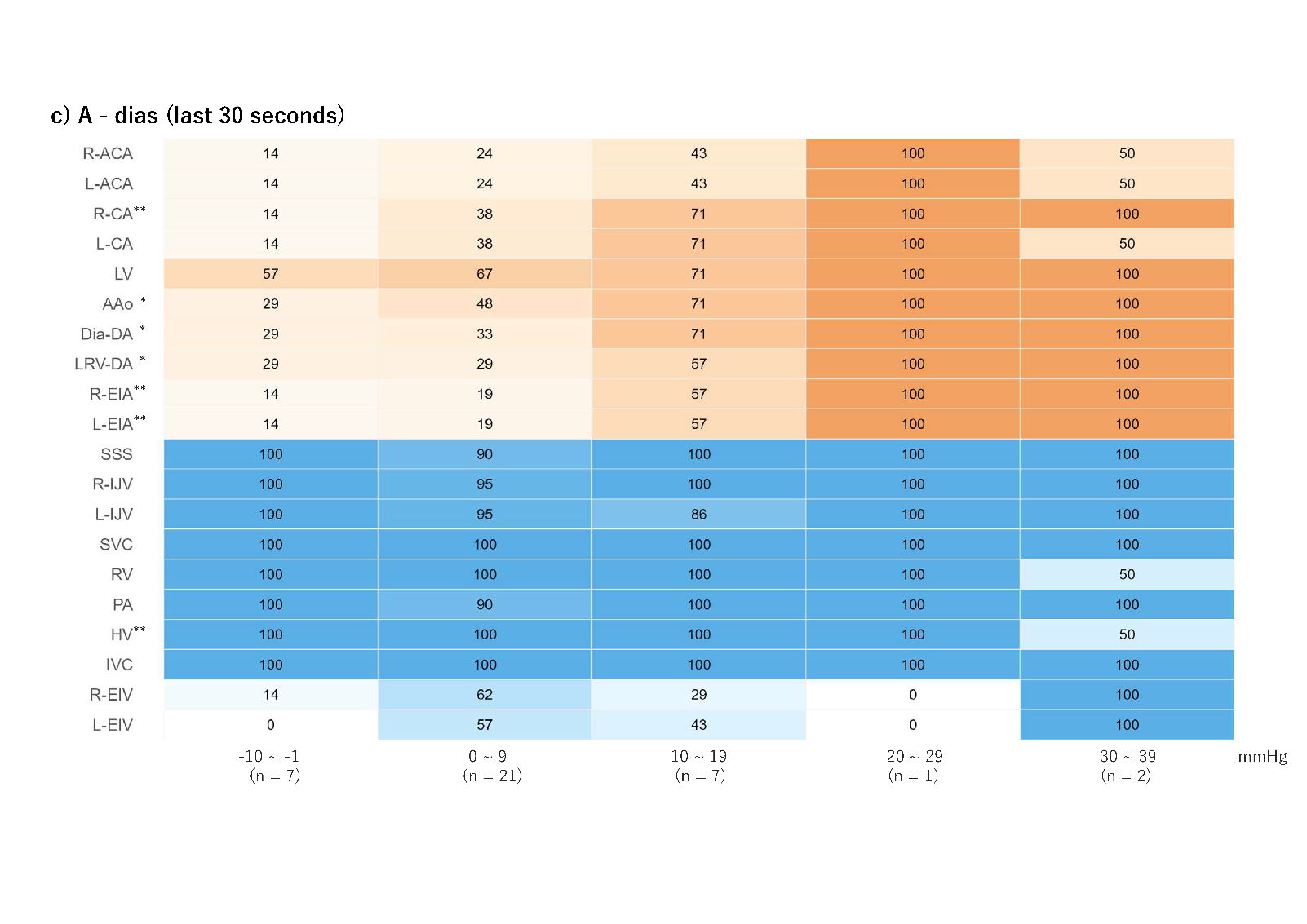
A dias

a) ΔSys A–V; b) A mean; c) A dias

Data are presented as the proportion (%) of patients with contrast enhancement in each vessel.

P values were calculated using the Cochran–Armitage test for trend　(*p for trend < 0.05; **p for trend < 0.01).

A dias, arterial diastolic pressure; A mean, arterial mean pressure; ΔSys A–V, difference between arterial and venous systolic pressures.

R-ACA, right-anterior cerebral artery; L-ACA, left-anterior cerebral artery; R-CA, right-common carotid artery; L-CA, left-common carotid artery; LV, left ventricle; AAo, ascending aorta; Dia-DA, descending aorta at the level of the diaphragm; LRV-DA, descending aorta at the level of the left renal vein; R-EIA, right-external iliac artery; L-EIA, left-external iliac artery; SSS, superior sagittal sinus; R-IJV, right-internal jugular vein; L-IJV, left-internal jugular vein; SVC, superior vena cava; RV, right ventricle; PA, pulmonary artery; HV, hepatic vein; IVC, inferior vena cava at the level of the left renal vein; R-EIV, right-external iliac vein; L-EIV, left-external iliac vein.

# eFigure 3. Heatmap analysis of the 2-minute period of each parameter obtained with each chest compression using the LUCAS 3


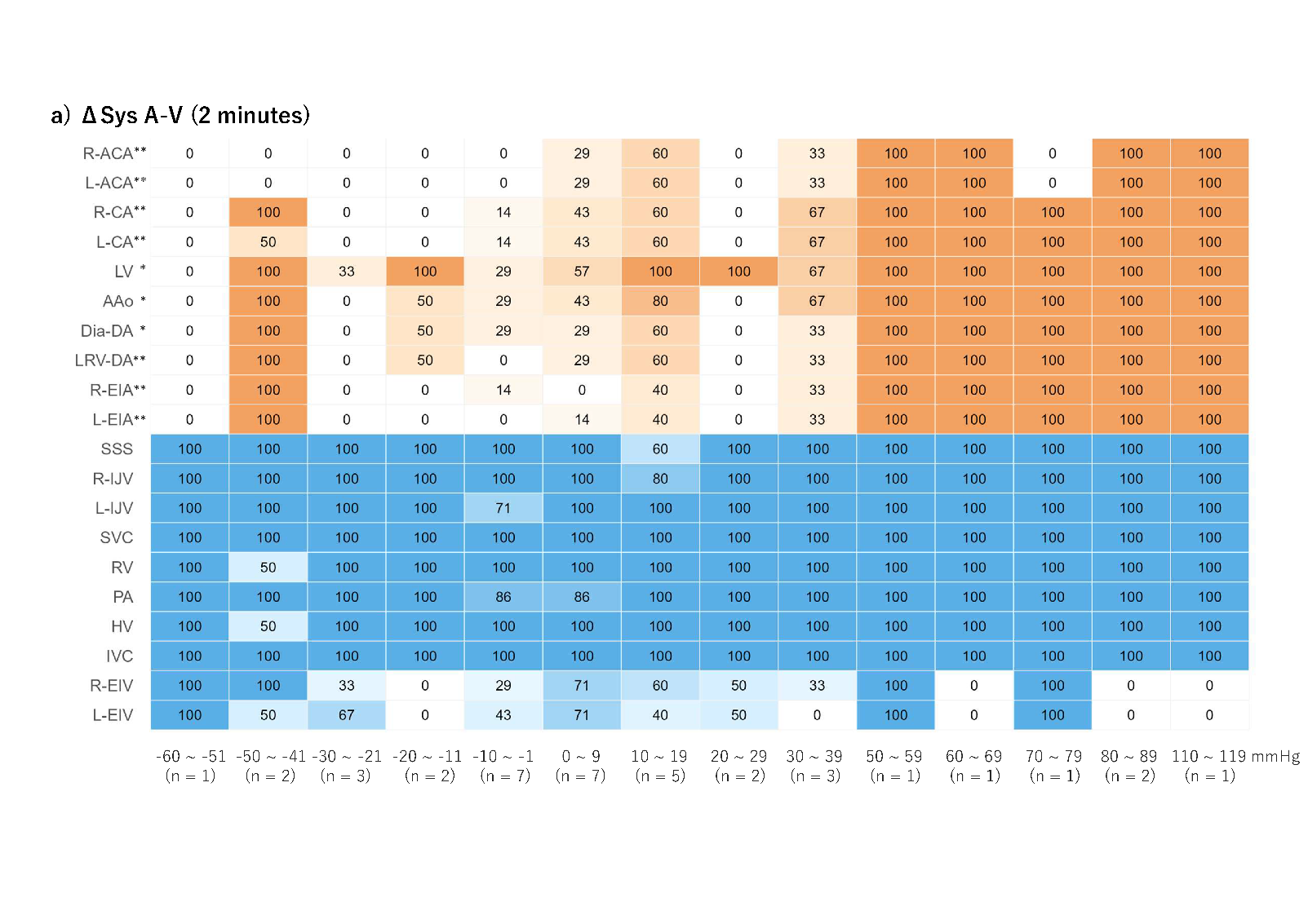
a) ΔSys A–V


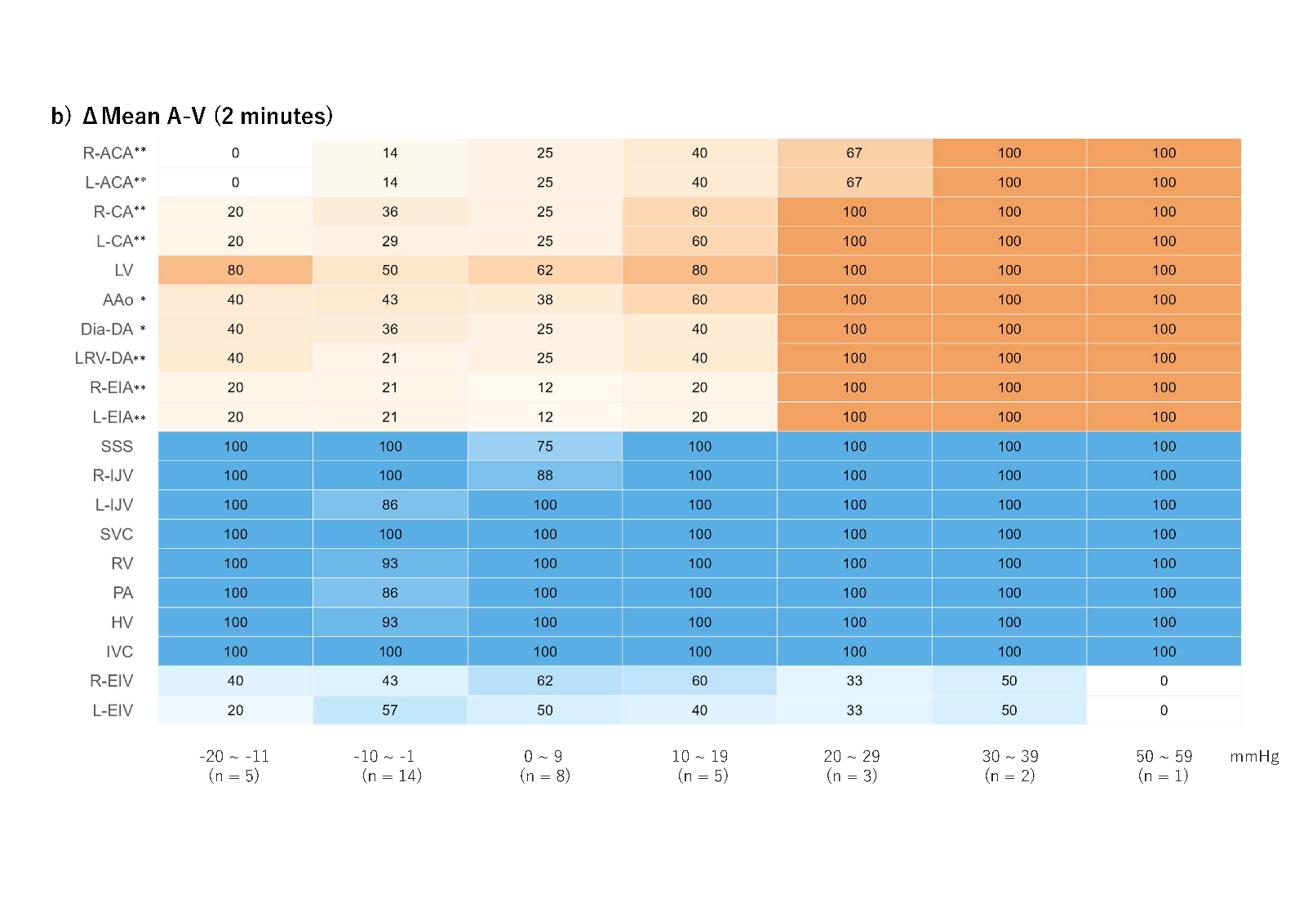
b) ΔMean A–V


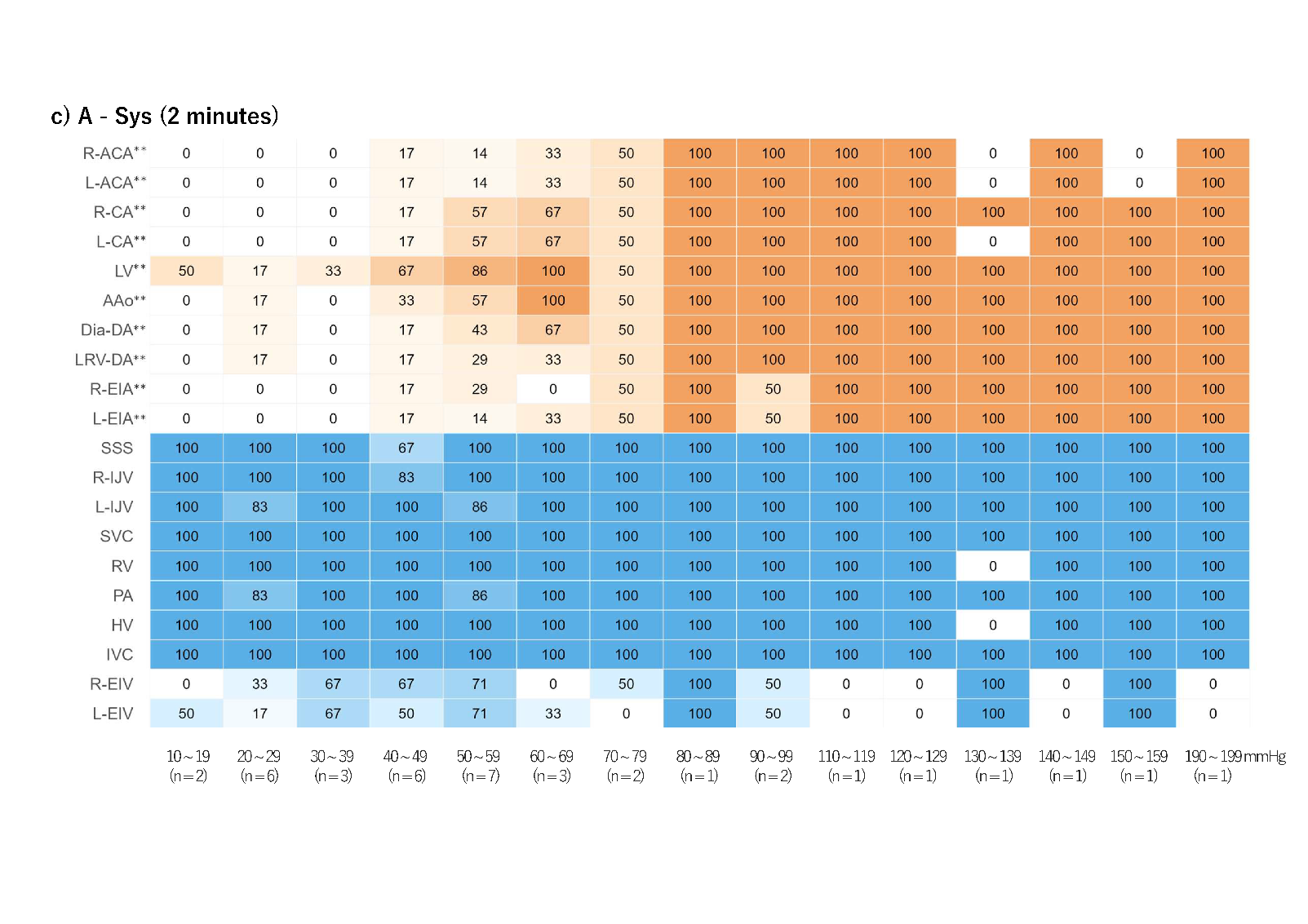
c) A sys


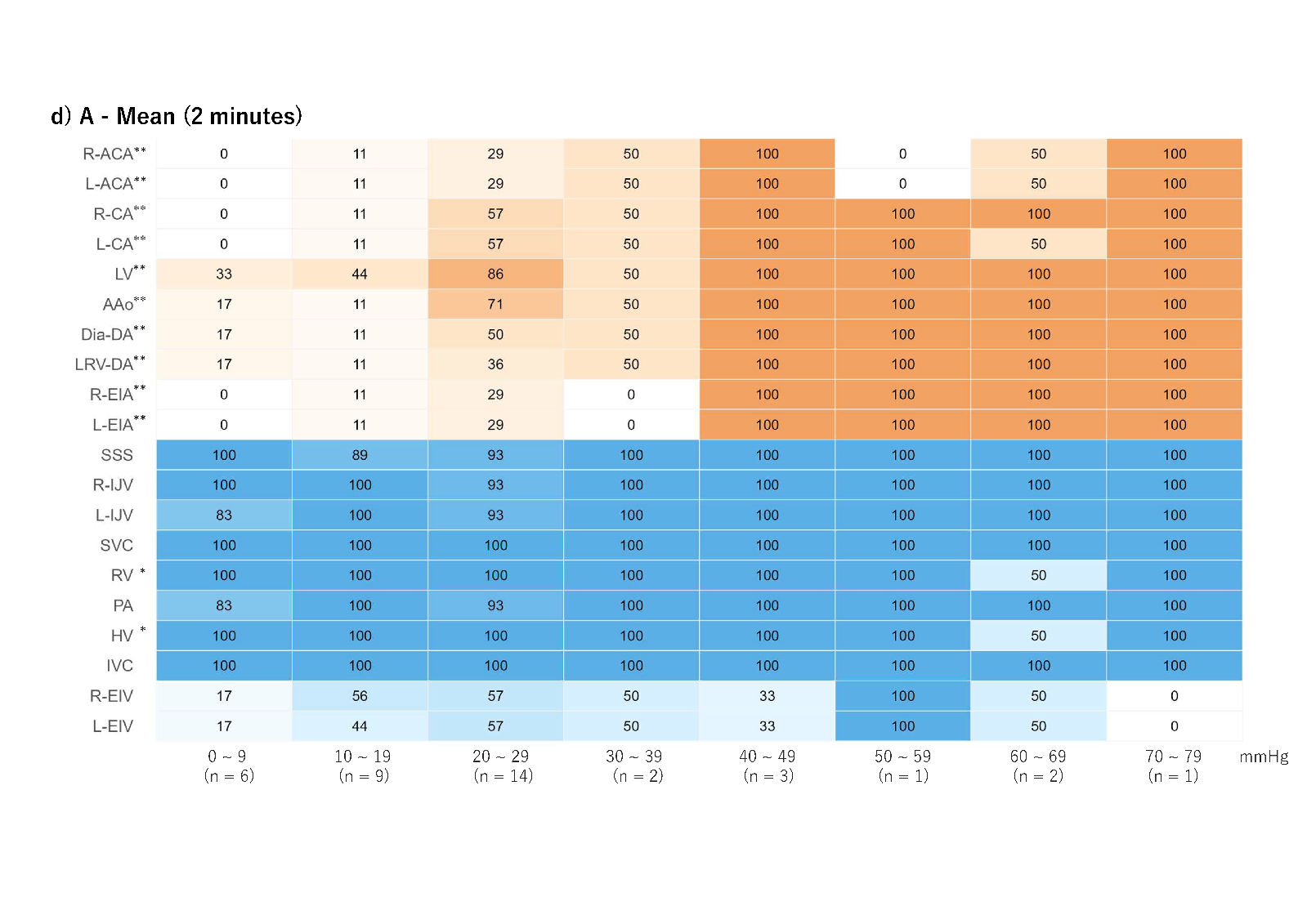
d) A mean


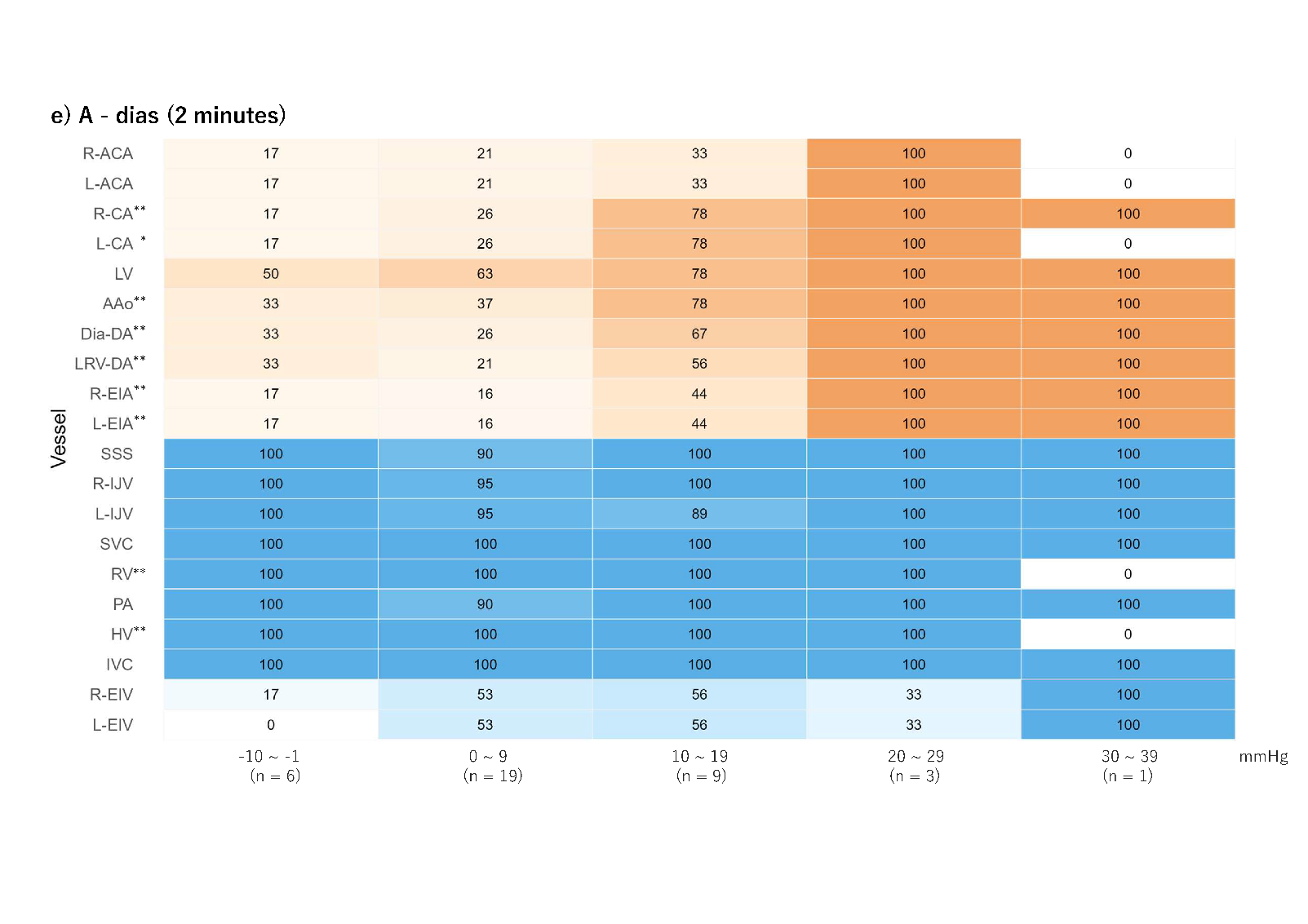
e) A dias

a) ΔSys A–V; b) ΔMean A–V; c) A sys; d) A mean; e) A dias

Data are presented as the proportion (%) of patients with contrast enhancement in each vessel.

P values were obtained using the Cochran–Armitage test for trend　(*p < 0.05; **p < 0.01).

A dias, arterial diastolic pressure; A mean, arterial mean pressure; A sys, arterial systolic pressure; ΔMean A–V, the difference between arterial and venous mean pressures; ΔSys A–V, the difference between arterial and venous systolic pressure.

R-ACA, right-anterior cerebral artery; L-ACA, left-anterior cerebral artery; R-CA, right-common carotid artery; L-CA, left-common carotid artery; LV, left ventricle; AAo, ascending aorta; Dia-DA, descending aorta at the level of the diaphragm; LRV-DA, descending aorta at the level of the left renal vein; R-EIA, right-external iliac artery; L-EIA, left-external iliac artery; SSS, superior sagittal sinus; R-IJV, right-internal jugular vein; L-IJV, left-internal jugular veins; SVC, superior vena cava; RV, right ventricle; PA, pulmonary artery; HV, hepatic vein; IVC, inferior vena cava at the level of the left renal vein; R-EIV, right-external iliac vein; L-EIV, left-external iliac vein.
